# Poly[[bis­{μ_3_-tris­[2-(1*H*-tetra­zol-1-yl)eth­yl]amine}copper(II)] bis­(perchlorate)]

**DOI:** 10.1107/S1600536810008998

**Published:** 2010-03-13

**Authors:** Franz Werner, Kenji Tokuno, Miki Hasegawa, Wolfgang Linert, Kurt Mereiter

**Affiliations:** a Aoyama-Gakuin University, College of Science and Engineering, Department of Chemistry and Biological Science, 5-10-1 Fuchinobe, Sagamihara, Kanagawa 229-8558, Japan; b Vienna University of Technology, Institute of Applied Synthetic Chemistry, Getreidemarkt 9/163, 1060 Vienna, Austria; c Vienna University of Technology, Institute of Chemical Technologies and Analytics, Getreidemarkt 9/164SC, A-1060 Vienna, Austria

## Abstract

In the title compound, {[Cu(C_9_H_15_N_13_)_2_](ClO_4_)_2_}_*n*_, the Cu^2+^ cation lies on an inversion center and is coordinated by the tetra­zole N^4^ atoms of six symmetry-equivalent tris­[2-(1*H*-tetra­zol-1-yl)eth­yl]amine ligands (*t* ^3^ *z*) in the form of a Jahn–Teller-distorted octa­hedron with Cu—N bond distances of 2.0210 (8), 2.0259 (8) and 2.4098 (8) Å. The tertiary amine N atom is stereochemically inactive. The cationic part of the structure, *viz.* [Cu(*t* ^3^ *z*)_2_]^2+^, forms an infinite two-dimensional network parallel to (100), in pockets of which the perchlorate anions reside. The individual networks are partially inter­locked and held together by C—H⋯O inter­actions to the perchlorate anions and C—H⋯N inter­actions to tetra­zole N atoms.

## Related literature

For a general procedure for the synthesis of tetra­zoles, see: Kamiya & Saito (1973 ▶). For the crystal structures of the *t* ^3^ *z* ligand and its complex with Cu(NO_3_)_2_, see: Hartdegen *et al.* (2009 ▶). For supra­molecular compounds made up of di-tetra­zolylalkanes, see: Liu *et al.* (2008 ▶); Yu *et al.* (2008 ▶). For Fe^2+^ spin-crossover complexes based on di-tetra­zolylalkanes, see: Grunert *et al.* (2004 ▶); Absmeier *et al.* (2006 ▶); Quesada *et al.* (2007 ▶); Bialonska *et al.* (2008 ▶). For a related structure, see: Werner *et al.* (2009 ▶).
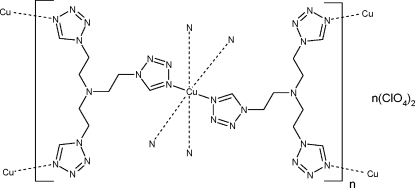


## Experimental

### 

#### Crystal data

[Cu(C_9_H_15_N_13_)_2_](ClO_4_)_2_
*M* *_r_* = 873.12
Triclinic, 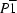

*a* = 8.5902 (3) Å
*b* = 9.4932 (4) Å
*c* = 11.8446 (5) Å
α = 69.233 (1)°
β = 74.652 (1)°
γ = 71.602 (1)°
*V* = 844.19 (6) Å^3^
*Z* = 1
Mo *K*α radiation
μ = 0.89 mm^−1^
*T* = 100 K
0.60 × 0.38 × 0.35 mm

#### Data collection

Bruker SMART APEX CCD diffractometer
Absorption correction: multi-scan (*SADABS*; Bruker, 2003 ▶) *T*_min_ = 0.86, *T*_max_ = 1.00
18905 measured reflections
5317 independent reflections
5160 reflections with *I* > 2σ(*I*)
*R*_int_ = 0.015

#### Refinement

*R*[*F* ^2^ > 2σ(*F* ^2^)] = 0.026
*wR*(*F* ^2^) = 0.072
*S* = 1.07
5317 reflections
250 parameters
H-atom parameters constrained
Δρ_max_ = 0.53 e Å^−3^
Δρ_min_ = −0.36 e Å^−3^

### 

Data collection: *SMART* (Bruker, 2003 ▶); cell refinement: *SAINT* (Bruker, 2003 ▶); data reduction: *SAINT*, *SADABS* and *XPREP* (Bruker, 2003 ▶); program(s) used to solve structure: *SHELXS97* (Sheldrick, 2008 ▶); program(s) used to refine structure: *SHELXL97* (Sheldrick, 2008 ▶); molecular graphics: *SHELXTL* (Sheldrick, 2008 ▶) and *Mercury* (Macrae *et al.*, 2006 ▶); software used to prepare material for publication: *SHELXL97* and *publCIF* (Westrip, 2010 ▶).

## Supplementary Material

Crystal structure: contains datablocks global, I. DOI: 10.1107/S1600536810008998/bq2196sup1.cif

Structure factors: contains datablocks I. DOI: 10.1107/S1600536810008998/bq2196Isup2.hkl

Additional supplementary materials:  crystallographic information; 3D view; checkCIF report

## Figures and Tables

**Table 1:** Hydrogen-bond geometry (Å, °)

*D*—H⋯*A*	*D*—H	H⋯*A*	*D*⋯*A*	*D*—H⋯*A*
C2—H2*A*⋯N10^i^	0.99	2.60	3.366 (2)	134
C4—H4⋯O2	0.95	2.33	3.191 (2)	151
C5—H5*B*⋯O1	0.99	2.58	3.557 (2)	168
C6—H6*A*⋯O4^ii^	0.99	2.54	3.459 (2)	154
C7—H7⋯O3^ii^	0.95	2.41	3.305 (2)	157
C8—H8*A*⋯N2^iii^	0.99	2.47	3.361 (2)	149
C8—H8*B*⋯O4^ii^	0.99	2.50	3.440 (2)	159
C8—H8*B*⋯O4^iv^	0.99	2.59	3.136 (2)	115

Symmetry codes: (i) 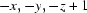; (ii) 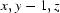; (iii) 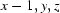; (iv) 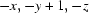.

**Fig. 1.**
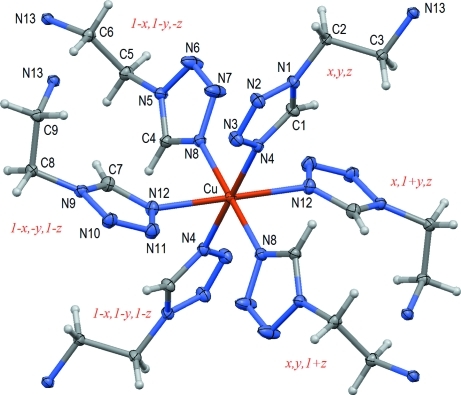
The coordination octahedron of Cu in (I) with incomplete t3z ligands. Displacement ellipsoids for the non-hydrogen atoms drawn at the 50% probability level. Symmetry codes for the t3z fragments are given in italics.

**Fig. 2.**
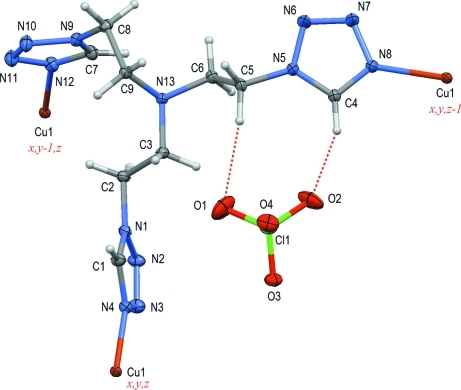
Asymmetric unit of (I) viewed along approximately perpendicular to the plane C3—C6—C9 of the t3z ligand. Displacement ellipsoids drawn at the 50% probability level. Symmetry codes of Cu atoms in italics. Two C—H···O hydrogen bonds shown as red broken lines.

**Fig. 3.**
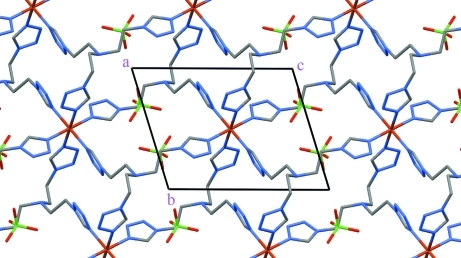
The two-dimensional coordination polymer in (I) extending parallel to (100) in a projection down the a-axis. H-atoms omitted for clarity.

**Fig. 4.**
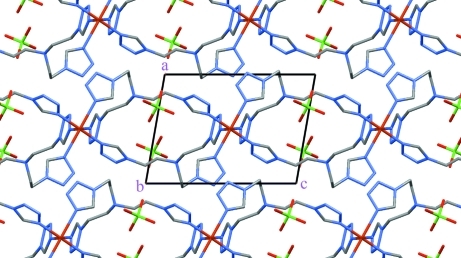
The two-dimensional coordination polymer in (I) extending parallel to (100) in a projection along the b-axis. H-atoms omitted for clarity.

**Fig. 5.**
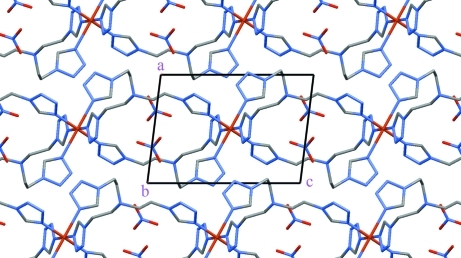
The structure of [Cu(t3z)2](NO3)2 (Hartdegen et al., 2009) in a view corresponding to Fig. 4 after shifting the coordinates by x' = x+1/2.

**Fig. 6.**
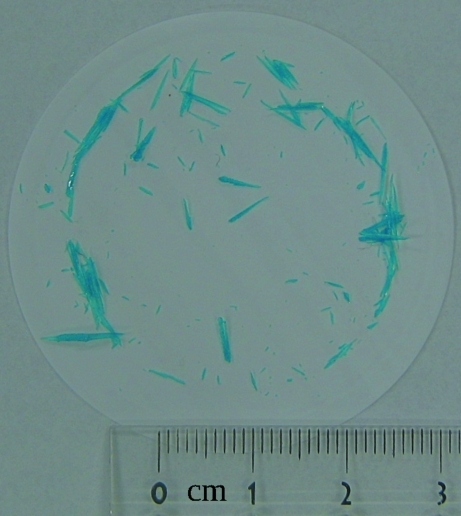
Crystals of [Cu(t3z)2](ClO4)2, as-grown from water.

## supplementary crystallographic information

### Comment

Polyfunctional molecules containing two or more 1*H*-tetrazol-1-yl groups linked by flexible spacer moieties are of considerable interest in supramolecular chemistry (e.g. Liu *et al.* 2008; Yu *et al.*, 2008) and in the construction of new Fe^2+^-based spin-crossover complexes (e.g. Grunert *et al.*, 2004; Absmeier *et al.*, 2006; Quesada *et al.*, 2007; Bialonska *et al.*, 2008). In continuation of previous studies (Werner *et al.*, 2009) the tris(2-(1*H*-tetrazol-1-yl)ethyl)-amine ligand (*t*^3^*z*) and the title compound were synthesized as described in the experimental section.

The title compound crystallizes in the triclinic space group P1 with one formula unit, [Cu(C_9_H_15_N_13_)_2_](ClO_4_)_2_, per unit cell. Copper lies on an inversion center (we selected *x*,*y*,*z* = 1/2, 1/2, 1/2 for Cu) and is coordinated by six symmetry equivalent *t*^3^*z* ligands via their 1*H*-tetrazole N4 atoms. The coordination figure about Cu (Fig. 1) is a Jahn-Teller distorted octahedron with four short Cu—N bonds (N4: 2 × 2.0259 (8) Å; N8: 2 × 2.0210 (8) Å) and two long Cu—N bonds (N12: 2 × 2.4098 (8) Å). The N—Cu—N angles are either 180° or deviate only by up to 2.20 (3)° from 90°. A view of the three-armed ligand with the three copper atoms bonded to it is shown in Fig. 2. The ligand adopts an unsymmetrical conformation with two ethyl groups in trans and one in gauche configuration (N1—C2—C3—N13 = 176.71 (8)°, N5—C5—C6—N13 = 170.74 (7)°, N9—C8—C9—N13 = 66.24 (10)°). It is obvious that the ligand is not chelating a copper atom but forms exclusively bridging links between each three of them. This is not unexpected because 1-alkyl-1*H*-tetrazoles coordinate transition metals generally via their N4 atoms (i.e. N4, N8 and N12 in the title compound) and the spacer length of four carbon plus one nitrogen atoms between two rigid tetrazole rings is too short to permit a reasonable chelation of a single metal centre. With this in mind it is clear that the structure of the title compound should be a coordination polymer. Instead of an expected three-dimensional network, the cationic part of the structure is an infinite two-dimensional coordination polymer extending parallel to (100), as shown in Figs. 3 and 4. The ClO_4_ anions are residing in pockets of this coordination polymer and are anchored *via* intra- as well as inter-layer C—H···O interactions (Table 1). Two of these interactions are depicted in Fig. 2.

Interestingly, the title compound turned out to be isostructural with [Cu(*t*^3^*z*)_2_](NO_3_)_2_ recently described by Hartdegen *et al.* (2009). This compound crystallizes similar to (I) in the triclinic space group *P*1 with *a* = 8.5850 (5) Å, *b* = 8.9606 (5) Å, *c* = 11.9532 (7) Å, *α* = 70.215 (5) Å, *β* = 76.919 (5)°, *γ* = 69.639 (5)°, *V* = 805.02 (8) Å^3^, and *Z* = 2 at *T* = 200 K. A view of this structure is presented in Fig. 5. After suitable origin selection the atomic coordinates of equivalent atomic positions of the [Cu(*t*^3^*z*)_2_] layers in the ClO_4_ and the NO_3_ salt differ for non-hydrogen atoms between 0 and 0.40 Å and on the average by 0.22 Å. The flat NO_3_ group is close in location to Cl1, O1, O2, and O3 in (I).

### Experimental

CAUTION! Tetrazoles and perchlorates are energetic compounds sensitive towards heat and impact. Proper precautions and care should be applied. The ligand tris(2-(1*H*-tetrazol-1-yl)ethyl)-amine, *t*^3^*z*, was prepared according to the general procedure of Kamiya & Saito (1973). A solution of 2.0 g tris(2-aminoethyl)-amine (13.7 mmol, Aldrich, 96%), 3.07 g sodium azide (47.2 mmol, Wako, min. 98.0%) and 9.12 g triethyl orthoformate (61.5 mmol, Sigma-Aldrich, 98%) in 120 ml glacial acetic acid (Kanto Chemical, 99.5%), was stirred for 3 h at a temperature of 343 - 353 K. After cooling to rt overnight the solvent was removed under reduced pressure. The solid residue was dissolved in 20 ml H_2_O, the solution was made alkaline (pH>11) by adding 100 ml of aqueous 4 N NaOH, and was then extracted with ethyl acetate. The combined organic layers were dried with sodium sulphate and the solvent was distilled off. The raw product was recrystallised from methanol yielding 178 mg (4.3%) of *t*^3^*z*. Elemental analysis (Micro Corder JM10, J-Science Lab): C (calculated 35.41%/found 35.85%), H (4.95/4.94), N (59.64/59.37). NMR (JEOL JNM-ECP 500): ^1^H(DMSO-d_6_) δ 3.01 (*t*, 6 H, CH_2_), 4.42 (*t*, 6H, CH_2_), 9.15 (*s*, 3 H, CH); ^13^C (DMSO-d_6_) δ 45.3 (CH_2_), 52.1 (CH_2_), 144.6 (CH).

Single crystals of the title complex, [Cu(*t*^3^*z*)_2_](ClO_4_)_2_, developed overnight from the combined solutions of 15.5 mg of Cu(ClO_4_)_2_^.^6H_2_O (0.041 mmol, Kanto Chemical) in 2.5 ml H_2_O and of 25.1 mg of *t*^3^*z *(0.082 mmol) in 5 ml H_2_O. Yield 28.2 mg (79%), blue needles (Fig. 6). Elemental analysis: C (calculated 24.76%/found 24.96%), H(3.46/3.52), N (41.71/42.62).

### Refinement

All H atoms were placed in calculated positions and thereafter treated as riding. *U*_iso_(H) = 1.2*U*_eq_(C) was used.

### Crystal data

**Table:**

[Cu(C_9_H_15_N_13_)_2_](ClO_4_)_2_	*Z* = 1
*M**_r_* = 873.12	*F*(000) = 447
Triclinic, *P*1	*D*_x_ = 1.717 Mg m^−^^3^
Hall symbol: -P 1	Mo *K*α radiation, λ = 0.71073 Å
*a* = 8.5902 (3) Å	Cell parameters from 7355 reflections
*b* = 9.4932 (4) Å	θ = 2.4–31.0°
*c* = 11.8446 (5) Å	µ = 0.89 mm^−^^1^
α = 69.233 (1)°	*T* = 100 K
β = 74.652 (1)°	Prism, blue
γ = 71.602 (1)°	0.60 × 0.38 × 0.35 mm
*V* = 844.19 (6) Å^3^	

### Data collection

**Table:**

Bruker SMART APEX CCD diffractometer	5317 independent reflections
Radiation source: normal-focus sealed tube	5160 reflections with *I* > 2σ(*I*)
graphite	*R*_int_ = 0.015
φ and ω scans	θ_max_ = 31.0°, θ_min_ = 2.4°
Absorption correction: multi-scan (*SADABS*; Bruker, 2003)	*h* = −12→12
*T*_min_ = 0.86, *T*_max_ = 1.00	*k* = −13→13
18905 measured reflections	*l* = −17→17

### Refinement

**Table:**

Refinement on *F*^2^	Primary atom site location: structure-invariant direct methods
Least-squares matrix: full	Secondary atom site location: difference Fourier map
*R*[*F*^2^ > 2σ(*F*^2^)] = 0.026	Hydrogen site location: inferred from neighbouring sites
*wR*(*F*^2^) = 0.072	H-atom parameters constrained
*S* = 1.07	*w* = 1/[σ^2^(*F*_o_^2^) + (0.0428*P*)^2^ + 0.303*P*] where *P* = (*F*_o_^2^ + 2*F*_c_^2^)/3
5317 reflections	(Δ/σ)_max_ = 0.001
250 parameters	Δρ_max_ = 0.53 e Å^−^^3^
0 restraints	Δρ_min_ = −0.36 e Å^−^^3^

### Special details

**Table:**

Geometry. All esds (except the esd in the dihedral angle between two l.s. planes) are estimated using the full covariance matrix. The cell esds are taken into account individually in the estimation of esds in distances, angles and torsion angles; correlations between esds in cell parameters are only used when they are defined by crystal symmetry. An approximate (isotropic) treatment of cell esds is used for estimating esds involving l.s. planes.
Refinement. Refinement of *F*^2^ against ALL reflections. The weighted *R*-factor wR and goodness of fit *S* are based on *F*^2^, conventional *R*-factors *R* are based on *F*, with *F* set to zero for negative *F*^2^. The threshold expression of *F*^2^ > σ(*F*^2^) is used only for calculating *R*-factors(gt) etc. and is not relevant to the choice of reflections for refinement. *R*-factors based on *F*^2^ are statistically about twice as large as those based on *F*, and *R*- factors based on ALL data will be even larger.

### Fractional atomic coordinates and isotropic or equivalent isotropic displacement parameters (Å^2^)

**Table:**

	*x*	*y*	*z*	*U*_iso_*/*U*_eq_	
Cu1	0.5000	0.5000	0.5000	0.01041 (5)	
N1	0.42188 (10)	0.17443 (9)	0.37722 (7)	0.01129 (14)	
N2	0.58575 (11)	0.11927 (10)	0.38325 (8)	0.01522 (16)	
N3	0.62835 (11)	0.21526 (10)	0.41688 (8)	0.01499 (15)	
N4	0.49368 (10)	0.33358 (9)	0.43272 (7)	0.01173 (14)	
N5	0.23080 (11)	0.34128 (10)	−0.15509 (8)	0.01398 (15)	
N6	0.19378 (16)	0.26607 (14)	−0.21840 (9)	0.0293 (2)	
N7	0.27560 (15)	0.30387 (14)	−0.32835 (9)	0.0271 (2)	
N8	0.36562 (11)	0.40410 (10)	−0.33836 (8)	0.01278 (15)	
N9	0.05800 (10)	−0.15980 (9)	0.33579 (7)	0.01167 (14)	
N10	−0.01294 (11)	−0.20876 (10)	0.45440 (8)	0.01605 (16)	
N11	0.09776 (11)	−0.32341 (11)	0.50942 (8)	0.01675 (16)	
N12	0.24167 (11)	−0.35052 (10)	0.42864 (8)	0.01439 (15)	
N13	0.19387 (10)	0.11244 (9)	0.17899 (7)	0.01133 (14)	
C1	0.36653 (12)	0.30591 (11)	0.40781 (9)	0.01306 (16)	
H1	0.2557	0.3687	0.4112	0.016*	
C2	0.33577 (12)	0.09397 (11)	0.33747 (9)	0.01269 (16)	
H2A	0.2419	0.0650	0.4026	0.015*	
H2B	0.4136	−0.0025	0.3230	0.015*	
C3	0.27031 (12)	0.19884 (11)	0.22024 (9)	0.01354 (16)	
H3A	0.1870	0.2925	0.2357	0.016*	
H3B	0.3629	0.2327	0.1562	0.016*	
C4	0.33503 (12)	0.42623 (11)	−0.22978 (8)	0.01214 (16)	
H4	0.3797	0.4914	−0.2088	0.015*	
C5	0.16668 (12)	0.31649 (11)	−0.02381 (9)	0.01354 (16)	
H5A	0.0434	0.3462	−0.0095	0.016*	
H5B	0.2054	0.3823	0.0063	0.016*	
C6	0.22876 (12)	0.14415 (11)	0.04621 (9)	0.01244 (16)	
H6A	0.1755	0.0811	0.0243	0.015*	
H6B	0.3505	0.1111	0.0195	0.015*	
C7	0.21343 (12)	−0.24693 (11)	0.32171 (9)	0.01360 (16)	
H7	0.2912	−0.2365	0.2472	0.016*	
C8	−0.02967 (11)	−0.02595 (11)	0.24720 (9)	0.01210 (16)	
H8A	−0.1514	−0.0119	0.2749	0.015*	
H8B	−0.0011	−0.0455	0.1665	0.015*	
C9	0.01822 (12)	0.12133 (11)	0.23426 (9)	0.01177 (15)	
H9A	−0.0525	0.2122	0.1825	0.014*	
H9B	−0.0021	0.1360	0.3161	0.014*	
Cl1	0.29526 (3)	0.69679 (3)	0.01464 (2)	0.01452 (6)	
O1	0.24265 (13)	0.56699 (11)	0.10773 (11)	0.0356 (2)	
O2	0.35608 (12)	0.66142 (13)	−0.10061 (10)	0.0316 (2)	
O3	0.42605 (10)	0.72936 (10)	0.04944 (8)	0.02103 (16)	
O4	0.15606 (10)	0.83116 (9)	0.00141 (8)	0.02162 (16)	

### Atomic displacement parameters (Å^2^)

**Table:**

	*U*^11^	*U*^22^	*U*^33^	*U*^12^	*U*^13^	*U*^23^
Cu1	0.01374 (8)	0.01175 (8)	0.00772 (8)	−0.00612 (6)	−0.00085 (5)	−0.00332 (5)
N1	0.0123 (3)	0.0121 (3)	0.0110 (3)	−0.0033 (3)	−0.0029 (3)	−0.0042 (3)
N2	0.0129 (4)	0.0158 (4)	0.0190 (4)	−0.0018 (3)	−0.0050 (3)	−0.0074 (3)
N3	0.0136 (4)	0.0153 (4)	0.0181 (4)	−0.0028 (3)	−0.0040 (3)	−0.0070 (3)
N4	0.0125 (3)	0.0134 (3)	0.0104 (3)	−0.0041 (3)	−0.0019 (3)	−0.0041 (3)
N5	0.0185 (4)	0.0172 (4)	0.0095 (3)	−0.0102 (3)	−0.0011 (3)	−0.0035 (3)
N6	0.0471 (7)	0.0421 (6)	0.0132 (4)	−0.0352 (5)	0.0045 (4)	−0.0111 (4)
N7	0.0437 (6)	0.0366 (5)	0.0132 (4)	−0.0315 (5)	0.0040 (4)	−0.0091 (4)
N8	0.0164 (4)	0.0140 (3)	0.0101 (3)	−0.0072 (3)	−0.0023 (3)	−0.0031 (3)
N9	0.0117 (3)	0.0124 (3)	0.0110 (3)	−0.0044 (3)	−0.0009 (3)	−0.0029 (3)
N10	0.0147 (4)	0.0186 (4)	0.0122 (4)	−0.0052 (3)	0.0001 (3)	−0.0022 (3)
N11	0.0160 (4)	0.0192 (4)	0.0129 (4)	−0.0049 (3)	−0.0015 (3)	−0.0026 (3)
N12	0.0149 (4)	0.0151 (4)	0.0128 (4)	−0.0031 (3)	−0.0029 (3)	−0.0040 (3)
N13	0.0128 (3)	0.0144 (3)	0.0091 (3)	−0.0069 (3)	−0.0012 (3)	−0.0036 (3)
C1	0.0133 (4)	0.0136 (4)	0.0144 (4)	−0.0036 (3)	−0.0026 (3)	−0.0063 (3)
C2	0.0162 (4)	0.0120 (4)	0.0126 (4)	−0.0052 (3)	−0.0049 (3)	−0.0037 (3)
C3	0.0180 (4)	0.0141 (4)	0.0117 (4)	−0.0083 (3)	−0.0051 (3)	−0.0019 (3)
C4	0.0144 (4)	0.0132 (4)	0.0099 (4)	−0.0059 (3)	−0.0017 (3)	−0.0029 (3)
C5	0.0174 (4)	0.0154 (4)	0.0083 (4)	−0.0066 (3)	0.0001 (3)	−0.0035 (3)
C6	0.0148 (4)	0.0138 (4)	0.0096 (4)	−0.0055 (3)	−0.0006 (3)	−0.0038 (3)
C7	0.0134 (4)	0.0141 (4)	0.0128 (4)	−0.0025 (3)	−0.0017 (3)	−0.0045 (3)
C8	0.0114 (4)	0.0120 (4)	0.0130 (4)	−0.0036 (3)	−0.0035 (3)	−0.0022 (3)
C9	0.0126 (4)	0.0120 (4)	0.0111 (4)	−0.0043 (3)	−0.0009 (3)	−0.0036 (3)
Cl1	0.01424 (10)	0.01325 (10)	0.01848 (11)	−0.00314 (7)	−0.00498 (8)	−0.00616 (8)
O1	0.0351 (5)	0.0191 (4)	0.0465 (6)	−0.0144 (4)	−0.0063 (4)	0.0039 (4)
O2	0.0295 (5)	0.0418 (5)	0.0332 (5)	0.0009 (4)	−0.0087 (4)	−0.0285 (4)
O3	0.0167 (3)	0.0320 (4)	0.0204 (4)	−0.0103 (3)	−0.0048 (3)	−0.0099 (3)
O4	0.0195 (4)	0.0178 (3)	0.0286 (4)	0.0029 (3)	−0.0097 (3)	−0.0106 (3)

### Geometric parameters (Å, °)

**Table:**

Cu1—N8^i^	2.0210 (8)	N13—C6	1.4579 (12)
Cu1—N8^ii^	2.0210 (8)	N13—C3	1.4617 (12)
Cu1—N4	2.0259 (8)	N13—C9	1.4656 (12)
Cu1—N4^iii^	2.0259 (8)	C1—H1	0.9500
Cu1—N12^iv^	2.4098 (8)	C2—C3	1.5215 (13)
Cu1—N12^v^	2.4098 (8)	C2—H2A	0.9900
N1—C1	1.3305 (12)	C2—H2B	0.9900
N1—N2	1.3500 (11)	C3—H3A	0.9900
N1—C2	1.4657 (12)	C3—H3B	0.9900
N2—N3	1.2915 (12)	C4—H4	0.9500
N3—N4	1.3599 (12)	C5—C6	1.5395 (13)
N4—C1	1.3249 (12)	C5—H5A	0.9900
N5—C4	1.3286 (12)	C5—H5B	0.9900
N5—N6	1.3500 (12)	C6—H6A	0.9900
N5—C5	1.4649 (12)	C6—H6B	0.9900
N6—N7	1.2892 (13)	C7—H7	0.9500
N7—N8	1.3614 (12)	C8—H8A	0.9900
N8—C4	1.3210 (12)	C8—H8B	0.9900
N8—Cu1^vi^	2.0209 (8)	C9—C8	1.5248 (13)
N9—C7	1.3332 (12)	C9—H9A	0.9900
N9—N10	1.3505 (11)	C9—H9B	0.9900
N9—C8	1.4668 (12)	Cl1—O1	1.4362 (9)
N10—N11	1.2982 (12)	Cl1—O4	1.4409 (8)
N11—N12	1.3630 (12)	Cl1—O3	1.4430 (8)
N12—C7	1.3262 (12)	Cl1—O2	1.4442 (10)
N12—Cu1^vii^	2.4098 (8)		
			
N8^i^—Cu1—N8^ii^	180.0	C3—C2—H2A	109.7
N8^i^—Cu1—N4	89.54 (3)	N1—C2—H2B	109.7
N8^ii^—Cu1—N4	90.46 (3)	C3—C2—H2B	109.7
N8^i^—Cu1—N4^iii^	90.46 (3)	H2A—C2—H2B	108.2
N8^ii^—Cu1—N4^iii^	89.54 (3)	N13—C3—C2	108.77 (7)
N4—Cu1—N4^iii^	180.0	N13—C3—H3A	109.9
N8^i^—Cu1—N12^iv^	88.33 (3)	C2—C3—H3A	109.9
N8^ii^—Cu1—N12^iv^	91.67 (3)	N13—C3—H3B	109.9
N4—Cu1—N12^iv^	92.20 (3)	C2—C3—H3B	109.9
N4^iii^—Cu1—N12^iv^	87.80 (3)	H3A—C3—H3B	108.3
N8^i^—Cu1—N12^v^	91.67 (3)	N8—C4—N5	108.02 (8)
N8^ii^—Cu1—N12^v^	88.33 (3)	N8—C4—H4	126.0
N4—Cu1—N12^v^	87.80 (3)	N5—C4—H4	126.0
N4^iii^—Cu1—N12^v^	92.20 (3)	N5—C5—C6	109.51 (8)
N12^iv^—Cu1—N12^v^	180.0	N5—C5—H5A	109.8
C1—N1—N2	108.62 (8)	C6—C5—H5A	109.8
C1—N1—C2	130.53 (8)	N5—C5—H5B	109.8
N2—N1—C2	120.81 (8)	C6—C5—H5B	109.8
N3—N2—N1	107.19 (8)	H5A—C5—H5B	108.2
N2—N3—N4	109.54 (8)	N13—C6—C5	113.54 (8)
C1—N4—N3	106.88 (8)	N13—C6—H6A	108.9
C1—N4—Cu1	130.20 (7)	C5—C6—H6A	108.9
N3—N4—Cu1	122.61 (6)	N13—C6—H6B	108.9
C4—N5—N6	108.65 (8)	C5—C6—H6B	108.9
C4—N5—C5	129.94 (8)	H6A—C6—H6B	107.7
N6—N5—C5	121.31 (8)	N12—C7—N9	108.87 (9)
N7—N6—N5	106.93 (9)	N12—C7—H7	125.6
N6—N7—N8	109.82 (9)	N9—C7—H7	125.6
C4—N8—N7	106.58 (8)	N9—C8—C9	110.61 (7)
C4—N8—Cu1^vi^	131.67 (7)	N9—C8—H8A	109.5
N7—N8—Cu1^vi^	121.62 (7)	C9—C8—H8A	109.5
C7—N9—N10	108.17 (8)	N9—C8—H8B	109.5
C7—N9—C8	129.90 (8)	C9—C8—H8B	109.5
N10—N9—C8	121.83 (8)	H8A—C8—H8B	108.1
N11—N10—N9	106.94 (8)	N13—C9—C8	111.13 (8)
N10—N11—N12	110.25 (8)	N13—C9—H9A	109.4
C7—N12—N11	105.77 (8)	C8—C9—H9A	109.4
C7—N12—Cu1^vii^	130.45 (7)	N13—C9—H9B	109.4
N11—N12—Cu1^vii^	120.86 (6)	C8—C9—H9B	109.4
C6—N13—C3	112.76 (7)	H9A—C9—H9B	108.0
C6—N13—C9	114.58 (7)	O1—Cl1—O4	109.30 (6)
C3—N13—C9	113.41 (8)	O1—Cl1—O3	109.62 (6)
N4—C1—N1	107.78 (8)	O4—Cl1—O3	109.26 (5)
N4—C1—H1	126.1	O1—Cl1—O2	109.78 (7)
N1—C1—H1	126.1	O4—Cl1—O2	109.38 (6)
N1—C2—C3	110.04 (7)	O3—Cl1—O2	109.49 (6)
N1—C2—H2A	109.7		
			
C1—N1—N2—N3	0.07 (11)	N2—N1—C1—N4	0.06 (11)
C2—N1—N2—N3	178.01 (8)	C2—N1—C1—N4	−177.61 (9)
N1—N2—N3—N4	−0.17 (11)	C1—N1—C2—C3	60.09 (13)
N2—N3—N4—C1	0.21 (11)	N2—N1—C2—C3	−117.34 (9)
N2—N3—N4—Cu1	174.39 (7)	C6—N13—C3—C2	−140.77 (8)
N8^i^—Cu1—N4—C1	−113.40 (9)	C9—N13—C3—C2	86.92 (10)
N8^ii^—Cu1—N4—C1	66.60 (9)	N1—C2—C3—N13	176.71 (8)
N12^iv^—Cu1—N4—C1	158.29 (9)	N7—N8—C4—N5	0.55 (12)
N12^v^—Cu1—N4—C1	−21.71 (9)	Cu1^vi^—N8—C4—N5	176.20 (7)
N8^i^—Cu1—N4—N3	73.91 (8)	N6—N5—C4—N8	−0.77 (12)
N8^ii^—Cu1—N4—N3	−106.09 (8)	C5—N5—C4—N8	175.60 (9)
N12^iv^—Cu1—N4—N3	−14.40 (8)	C4—N5—C5—C6	−115.73 (11)
N12^v^—Cu1—N4—N3	165.60 (8)	N6—N5—C5—C6	60.24 (13)
C4—N5—N6—N7	0.68 (15)	C3—N13—C6—C5	−59.54 (10)
C5—N5—N6—N7	−176.05 (11)	C9—N13—C6—C5	72.20 (10)
N5—N6—N7—N8	−0.34 (16)	N5—C5—C6—N13	170.74 (7)
N6—N7—N8—C4	−0.13 (14)	C6—N13—C9—C8	79.11 (9)
N6—N7—N8—Cu1^vi^	−176.32 (9)	C3—N13—C9—C8	−149.46 (8)
C7—N9—N10—N11	−0.45 (11)	C7—N9—C8—C9	−80.18 (12)
C8—N9—N10—N11	−177.15 (8)	N10—N9—C8—C9	95.73 (10)
N9—N10—N11—N12	0.29 (11)	N13—C9—C8—N9	66.24 (10)
N10—N11—N12—C7	−0.02 (11)	N11—N12—C7—N9	−0.27 (11)
N10—N11—N12—Cu1^vii^	162.54 (7)	Cu1^vii^—N12—C7—N9	−160.50 (7)
N3—N4—C1—N1	−0.17 (11)	N10—N9—C7—N12	0.45 (11)
Cu1—N4—C1—N1	−173.74 (6)	C8—N9—C7—N12	176.79 (9)

Symmetry codes: (i) −*x*+1, −*y*+1, −*z*; (ii) *x*, *y*, *z*+1; (iii) −*x*+1, −*y*+1, −*z*+1; (iv) −*x*+1, −*y*, −*z*+1; (v) *x*, *y*+1, *z*; (vi) *x*, *y*, *z*−1; (vii) *x*, *y*−1, *z*.

### Hydrogen-bond geometry (Å, °)

**Table:**

*D*—H···*A*	*D*—H	H···*A*	*D*···*A*	*D*—H···*A*
C2—H2A···N10^viii^	0.99	2.60	3.366 (2)	134
C4—H4···O2	0.95	2.33	3.191 (2)	151
C5—H5B···O1	0.99	2.58	3.557 (2)	168
C6—H6A···O4^vii^	0.99	2.54	3.459 (2)	154
C7—H7···O3^vii^	0.95	2.41	3.305 (2)	157
C8—H8A···N2^ix^	0.99	2.47	3.361 (2)	149
C8—H8B···O4^vii^	0.99	2.50	3.440 (2)	159
C8—H8B···O4^x^	0.99	2.59	3.136 (2)	115

Symmetry codes: (viii) −*x*, −*y*, −*z*+1; (vii) *x*, *y*−1, *z*; (ix) *x*−1, *y*, *z*; (x) −*x*, −*y*+1, −*z*.
